# Draft genome and alcohol dehydrogenase dataset of thermoanaerobacter uzonensis bacterium strain AK85

**DOI:** 10.1016/j.dib.2025.112192

**Published:** 2025-10-27

**Authors:** Clay A. Abraham, Eva Maria Ingvadottir, Kevin M. Bradley, Sean Michael Scully, Johann Orlygsson, Derek Dube, Steven A. Benner

**Affiliations:** a Foundation for Applied Molecular Evolution#, Alachua, FL, USA; b Department of Biology, University of Saint Joseph$, West Hartford, CT, USA; c Faculty of Natural Resource Sciences, University of Akureyri†, Akureyri, Iceland

**Keywords:** *Thermoanaerobacter uzonensis*, Prokka genome annotation, Alcohol dehydrogenase

## Abstract

*Thermoanaerobacter uzonensis* strain AK85 belongs to the *Thermoanaerobacter* genus, which comprises rod-shaped, Gram-positive, thermophilic, obligate anaerobic bacteria. Members of this genus exhibit unique fermentation qualities, such as prolific ethanol production, and can generate longer-chain alcohols from carbohydrate and amino acid sources. Here we present the draft genome sequence of *Thermoanaerobacter uzonensis* strain AK85, which was previously isolated from a hot spring in Graensdalur in Southwestern Iceland. The genome was sequenced with a 150 bp paired-end library on a MGISEQ-2000. The assembled genome comprises 2,577,794 bp and a GC ratio of 33.69 %. With an ANI of 96.9 % strain AK85 was determined to be a strain of *Thermoanaerobacter uzonensis*. Annotation was conducted with Prokka which revealed 41 enzymes related to carbohydrate, amino acid, and carboxylic acid metabolism. The genomic dataset establishes the biotechnological capacity and potential of strain AK85 for the production of alcohols and other bio-manufactured products. Further, the genomic dataset is coupled with a cofactor and substrate analysis of the three detected alcohol dehydrogenases. These enzymes were assessed via a lysate based colorimetric assay with NAD^+^ and NADP^+^. Under these conditions the native alcohol dehydrogenases are able to oxidize long chain primary alcohols such as 1-octanol and benzyl alcohol. The reads and assembled draft genome of AK85 were deposited into SRA and NCBI under Bioproject PRJNA1108289, Genbank JBDHNK000000000, and Biosample SAMN41233939.

Specifications Table

**Table:** 

Subject	Biology
Specific subject area	Genomics and Molecular Biology
Type of data	Tables, Figures, and Charts
Data collection	Genomic DNA of *Thermoanaerobacter uzonensis* strain AK85 was extracted and sequenced using the MGISEQ-2000 platform. The De novo assembly was generated with SKESA (v2.5.0). QUAST (v5.2.0), CheckM (v0.9.7), and BUSCO (v6.0.0) was used to assess assembly quality and associated metrics. Prokka (v1.2.0) was used to annotate the genome. Alcohol Dehydrogenase activity lysate data was obtained by combining lysate and a colormetric assay solution and 590 nm absorbance was recorded for 1 hour on a Bioscreen C microplate reader.
Data source location	Foundation for Applied Molecular Evolution and University of Akureyri, Iceland
Data accessibility	Repository Name: National Center for Biotechnology Information (NCBI) Data Identification Number: The BioSample number is SAMN41233939; The BioProject is PRJNA1108289; The Genome Accession number is JBDHNK000000000; and the Genome Assembly number is ASM5083913v. Direct URL: https://www.ncbi.nlm.nih.gov/datasets/genome/GCA_050839135.1/https://www.ncbi.nlm.nih.gov/bioproject/?term=PRJNA1108289 Repository Name: Sequencing Read Archive (SRA) Data Identification Number: SRX29078513 Direct URL: https://www.ncbi.nlm.nih.gov/sra/SRX29078513[accn] Repository Name: Mendeley Data Repository Data Identification Number: 10.17632/8zcv8kzg6j.1 Direct URL: https://data.mendeley.com/datasets/8zcv8kzg6j/1
Related research article	J.E. Jessen, J. Orlygsson, Production of ethanol from sugars and lignocellulosic biomass by Thermoanaerobacter J1 iIsolated from a hot spring in Iceland,. J. of Biomed. and Biotechnology. (2012):1-7. https://doi.org/10.1155/2012/186982.

## 1 Value of the Data

• Based on genomic annotation, Thermoanaerobacter uzonensis strain AK85 has a PTS lactose/cellobiose transporter and a cellulase glycosyl hydrolase. Therefore, AK85 exhibits potential as a biotechnology platform for the renewable synthesis of alcohols from complex biomass.
• The AK85 genomic dataset enables researchers to perform a comparative genomic analyses to T. uzonensis and other diverse members of the Thermoanaerobacter genus.
• The annotation dataset aids researchers investigating thermophilic anaerobic extremophiles and their micro-ecological role in extreme anaerobic environments.
• The alcohol dehydrogenase lysate analysis demonstrates the broad specificity of alcohol oxidation in the presence of NAD+ and NADP+ as cofactors.
• AK85 and other members of the Thermoanaerobacter genus are naturally competent thus this cell strain could be used to create a thermophilic anaerobic protein expression platform.

## 2 Background

*Thermoanaerobacter uzonensis* strain AK85 (AK85) is a thermophilic anaerobic bacterium that was isolated from hot spring sediment in Graensdalur, Iceland [1]. The strain was isolated from anaerobic sediment via serial dilution in Basal Mineral (BM) medium [1,2]. While previous studies have conducted 16S rRNA and fermentation end product analysis, the following work presents the sequencing, assembly, and annotation of the AK85 genome. Currently, the *Thermoanaerobacter* genus contains 18 identified species and numerous unclassified strains to which AK85 belongs to. The genus is highly diverse with strains able to utilize a variety of carbon sources and amino acids for fermentation [[1], [2], [3], [4]]. Further, some strains within the genus produce alcohols including propanol, butanol and other long chain alcohols.

## 3 Data Description

**Fig. 1:**
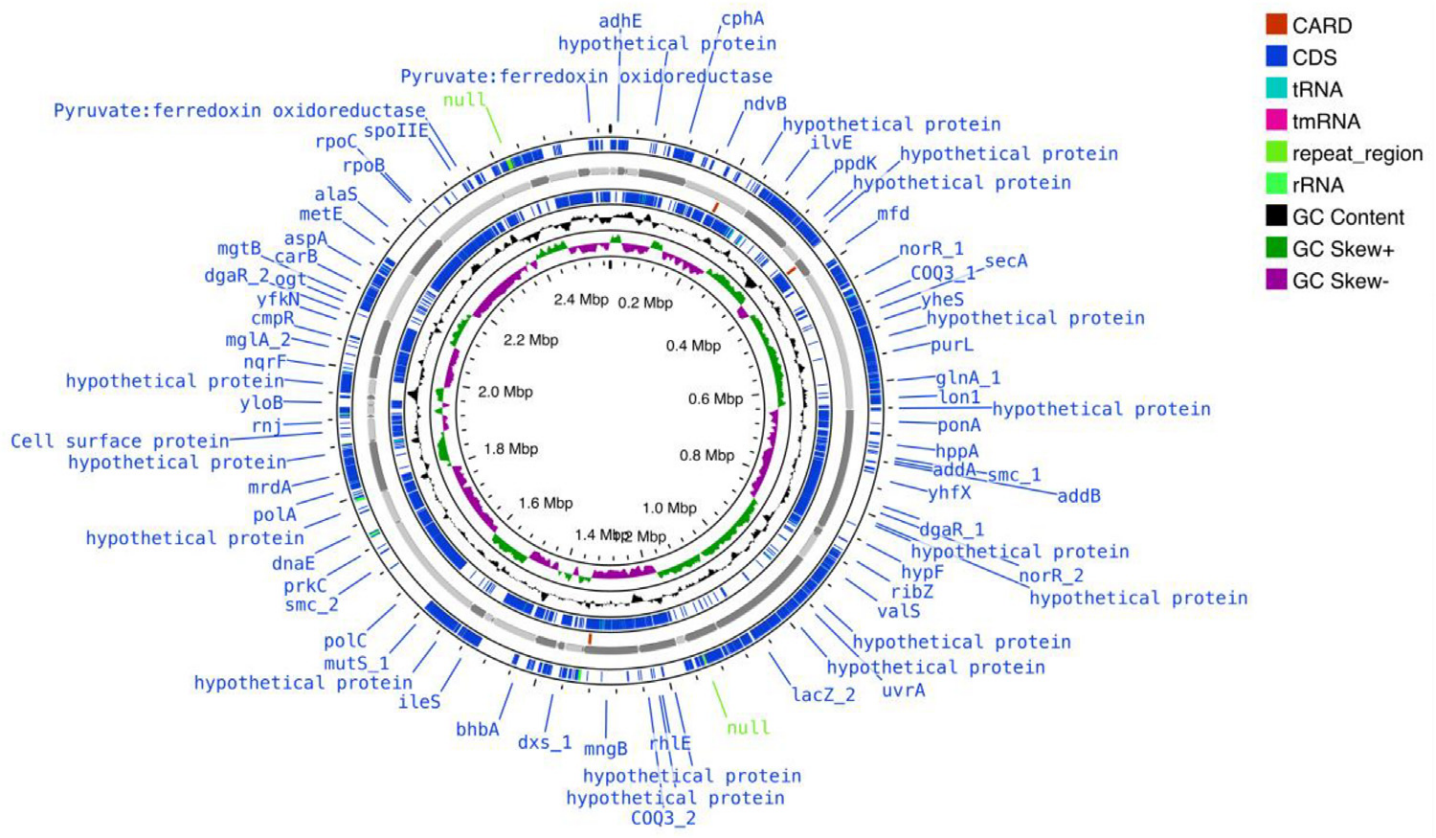
Above is the genome map of *T. uzonensis* strain AK85 created in Proskee (website). Annotations are color coded according to the legend in the top right. The two blue outer circles contain the CDS regions which include the tRNA, tmRNA, rRNA and repeat regions. The next inner circle displays the contigs in grey, followed by the vancomycin resistance genes identified with CARD. The 2nd most inner circle is a representation of GC content in green and purple and GC skew in black. The inner most circle displays the genome size in Mbp.

**Fig. 2:**
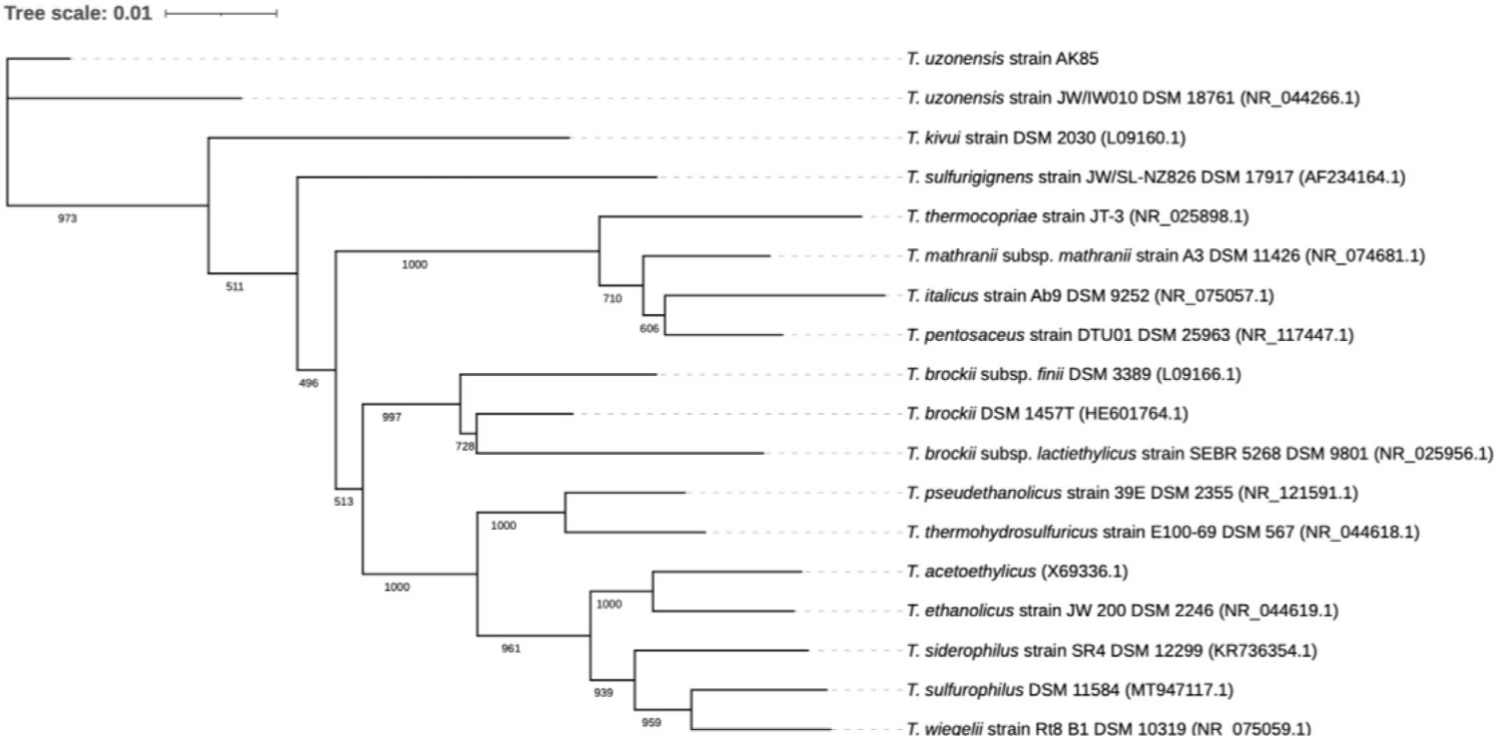
This figure contains the aligned 16S rRNA sequences of 17 type strains within the *Thermoanaerobacter* genus assembled into a distance based phylogenetic tree with 1000 bootstrap replications using FastME. The raw bootstrap values out of 1000 are displayed at each branch node. A Tree scale of 0.01 represents a 1 % basepair difference corresponding to the branch lengths. As observed in the figure above, strain AK85 at the top of the tree clusters closely with *T. uzonensis.* Genbank accession no. are listed for each entry in ().

Here we present the draft genomic sequencing and annotation data of *T. uzonensis* strain AK85. Included is a summary of the sequencing and assembly data in Table 1. Similarly, a genome map created in Proskee and annotated with Prokka, using default parameters in both instances, for examining relevant genes for generating relevant bio-manufacturing targets is presented in Fig. 1 [5,6]. The assembled genome contains 63 contigs, 2,577,794 bp, a N50 of 88,182, a L50 of 9, a GC ratio of 33.69 %, a Complete BUSCO of 98.3 %, and a Partial BUSCO of 1.7 % as observed in Table 1. The draft genome contains 2528 coding sequences (CDS), 53 transfer-RNAs (tRNAs), 1 transfer messenger-RNA (tmRNA), and 4 ribosomal-RNAs (rRNAs) as seen in Fig. 1. The Average Nucleotide Identity (ANI) was determined to be 96.9 % compared to *T. uzonensis* DSM 18761 [7]. Moreover, the 16S rRNA sequences from AK85 clustered with *T. uzonensis* when compared to 16 other members of the *Thermoanaerobacter* genus as seen in Fig. 2*.*

**Table 1:** Displays the raw sequencing data and post SKESA assembly QUAST report of *T. uzonensis* strain AK85. Notably the assembly contained 63 total contigs, a total length of 2.57 Mbp, a N50 of 88,182, a L50 of 9, zero N content, and a Complete BUSCO of 98.3 %.

MiSeq Sequencing Data			
**Clean Reads**	4,072,726		
**Clean Bases**	1,221,817,800		
**Q20 (%)**	96.39		
**Q30 (%)**	90.10		
**Genome Coverage**	507x		
**SKESA Assembly QUAST Report**			
**Number of Contigs**	63		
**Total Length (bp)**	2,577,794		
**Largest Contig (bp)**	243,850		
**N50 (bp)**	88,182		
**N90 (bp)**	30,736		
**L50 (bp)**	9		
**L90 (bp)**	28		
**GC Content (%)**	33.69		
**# Ν’s**	0		
**# N’s Per 100 kbp**	0		
**CheckM Completeness (%)**	96.5		
**CheckM Contamination (%)**	1.5		
**Complete BUSCO (%)**	98.3		
**Partial BUSCO (%)**	1.7		
Enzyme	Length (bp)	Gene Name	EC number
Carboxylic Acid Metabolism			
Butyrate kinase 2	1068	*buk2_1*	2.7.2.7
Butyrate kinase 2	1062	*buk2_2*	2.7.2.7
Putative butyrate:acetyl-CoA coenzyme A-transferase	1296		2.8.3.-
Butyrate–acetoacetate CoA-transferase subunit B	666	*ctfB*	2.8.3.9
Butyrate–acetoacetate CoA-transferase subunit A	666	*ctfA*	2.8.3.9
Alcohol Dehydrogenases			
Aldehyde-alcohol dehydrogenase	2619	*adhE*	1.1.1.1/1.2.1.10
Long-chain primary alcohol dehydrogenase AdhA	1200	*adhA*	1.1.1.2
Long-chain-alcohol dehydrogenase 2	1170	*adh2*	1.1.1.192

The Card annotation within Proskee identified multiple gene clusters that are relevant for antibiotic resistance [8], including three genes conferring natural vancomycin resistance as displayed in Fig. 1. Additionally, the genome was manually searched for alcohol dehydrogenases, particularly with butanol and propanol formation in mind as each compound is industrially relevant. These results are listed in Table 2. Furthermore, genes related to carbohydrate utilization, amino acid fermentation, sulfite cycling, ethanol fermentation, esterases, and lipases can be viewed in Supplementary Tables 1.1 and 1.2. Of special note is the strain’s ability to utilize mono-, di-, oligo-, and polysaccharides as a carbon source. This includes monosaccharides such as glucose, trehalose, and xylose, and more complex carbohydrates such as cellobiose and starch, which can be derived from renewable lignocellulosic biomass which in turn has implications for biotechnology applications [9].

**Table 2:** This table lists the annotated genes by Prokka related to butanol and propanol synthesis with the correlated Gene Name, EC number for enzymatic activity, and the length of the gene.

Enzyme	Length (bp)	Gene Name	EC number
**Carboxylic Acid Metabolism**			
Butyrate kinase 2	1068	*buk2_1*	2.7.2.7
Butyrate kinase 2	1062	*buk2_2*	2.7.2.7
Putative butyrate:acetyl-CoA coenzyme A-transferase	1296		2.8.3.-
Butyrate–acetoacetate CoA-transferase subunit B	666	*ctfB*	2.8.3.9
Butyrate–acetoacetate CoA-transferase subunit A	666	*ctfA*	2.8.3.9
**Alcohol Dehydrogenases**			
Aldehyde-alcohol dehydrogenase	2619	*adhE*	1.1.1.1/1.2.1.10
Long-chain primary alcohol dehydrogenase AdhA	1200	*adhA*	1.1.1.2
Long-chain-alcohol dehydrogenase 2	1170	*adh2*	1.1.1.192

As observed in Table 2, strain AK85 contains several enzymes related to butyrate and butanol synthesis. These include butyrate kinases, butyrate–acetoacetate CoA-transferases, two propionyl-CoA carboxylase β subunits and three alcohol dehydrogenases. The alcohol dehydrogenase enzymes include a bifunctional aldehyde-alcohol dehydrogenase (AdhE) and two long chain alcohol dehydrogenases (Adh). Each Adh was annotated as AdhA and Adh2 respectively. All of the aforementioned enzymes are theoretically involved in the interconversion of carboxylic acids to their corresponding alcohols under fermentation conditions previously investigated by Scully & Orlygsson [2].

To summarize, the annotation of the AK85 genome reveals a robust thermophilic anaerobe that has potential in multiple bio-manufacturing roles as indicated by its ability to utilize a variety of carbohydrates.

**Fig. 3:**
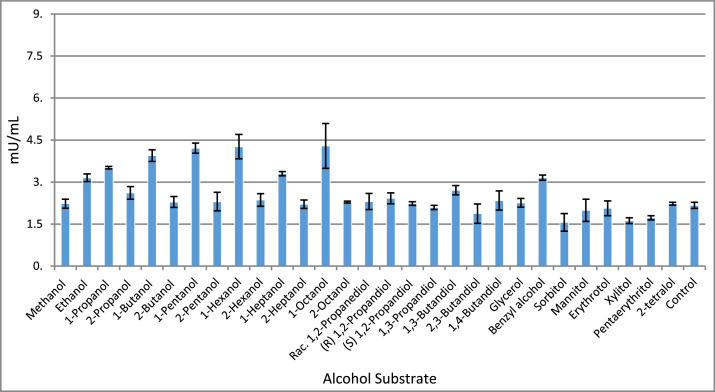
This chart displays the oxidation of alcohol compounds in the presence of NAD+. Each reading was done in technical triplicate the error bars representing the standard deviation. As seen above, multiple primary alcohols are preferred over their secondary alcohol counterpart. Interestingly, very large alcohols such as benzyl alcohol and 1-octanol each show elevated levels of conversion. Abbreviation: racemic (Rac.)

**Fig. 4:**
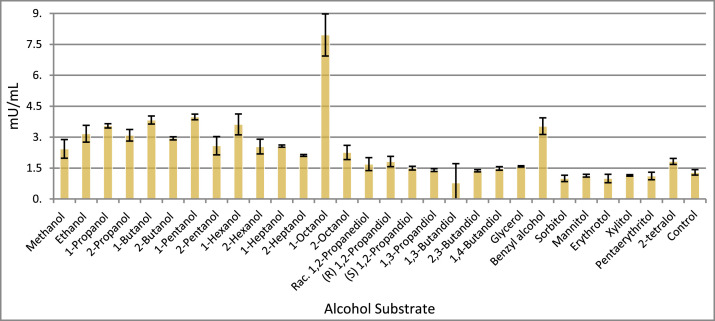
The chart above is the ADH lysate analysis of AK85 with multiple alcohol compounds and using NADP^+^ as the primary cofactor. Each reading was done in technical triplicate with the error bars representing the standard deviation. As observed in the data above, with NADP^+^ 1-octanol and benzyl alcohol are both the preferred substrates. However, there is increased activity above the negative control with several of the shorter primary alcohols including 1-Propanol, 1-Butanol, 1-Pentanol, and 1-Hexanol. Abbreviation: racemic (Rac.)

While the fermentation capacities of strain AK85 have been previously investigated, laying the foundation for its genome sequence, the specific activities of the detected AdhE and Adh enzymes remain unexplored in the literature. Alcohol dehydrogenases are of particular interest due to their applications in biotechnology and biomanufacturing. Following annotation of the Adh enzymes with Prokka, their amino acid sequences were analyzed using the InterPro database [10], which confirmed that each Adh gene contains a NAD^+^-binding site, suggesting NAD^+^ as the preferred cofactor. Notably, InterPro classified the Adh2 sequence as a butanol dehydrogenase. To evaluate the catalytic activity of these Adh enzymes with various alcohol substrates, a colorimetric lysate assay was performed using NAD^+^ or NADP^+^ as cofactors, with results shown in Fig. 3, Fig. 4, respectively. The lysate assay reveals the selectivity of all three alcohol dehydrogenases at their native expression levels under saturating NAD^+^ or NADP^+^ conditions.

In summary, the lysate assay included primary, secondary, diol, and sugar alcohol compounds. Interestingly, while the Adh enzymes were detected to have NAD^+^ specific binding sites, NADP^+^ was also an effective cofactor for enzymatic activity as seen in Fig. 4. Further, the data suggests that the Adh activities with NAD^+^ prefer primary alcohols and benzyl alcohol as substrates. While with NADP^+^, specificity is widened to include secondary alcohols, diols and 2-tetralol. A similar trend has been reported for other members of the genus, including *T. ethanolicus, T. pseudethanolicus, T. brockii, T. finnii* and *Thermoanaerobacter* sp. strain X514, where higher activity on secondary substrates is observed to be NADP^+^ dependent [11].

The lysate data indicates that a very broad substrate specificity of the Adh enzymes is essential for the interconversion of aldehydes and alcohols, which would further suggest that AK85 contains several biotechnology applications for the production of bio-manufactured alcohols, aldehydes or carboxylic acids under the correct conditions.

## 4 Experimental Design, Materials and Methods

### 4.1 DNA extraction

A culture of *T. uzonensis* strain AK85, previously isolated from a hot spring sediment in SW Iceland [1], was inoculated in 7.5 mL of BM medium from a freezer stock (consisting of 30 % glycerol in BM and culture broth in equal amounts). BM media was comprised of 50 mM phosphate buffer pH 7.0, 2 g/L yeast extract, 5.60 mM NH4Cl, 5.13 mM NaCl, 0.75 mM CaCl2·2H2O, 0.49 mM MgCl2·6H2O, 9.5 mM NaHCO3, 20 mM glucose, 2 µM resazurin, 3.52 mM L-cysteine-HCl, 0.022 µM Na2S·9H2O, 1 mL trace element solution, and 1 mL vitamin solution [1,2]. The culture was grown under strict anaerobic conditions with N_2_ gas for 48 h at 65 °C without shaking. The culture was transferred into a 15 mL tube and pelleted at 3.5k x g for 15 min at 4 °C. The supernatant was decanted and the cell pellet then stored at −80 °C. For extraction, the pellet was thawed and treated with an SDS lysis solution for 30 minutes at 37°C [12]. Proceeding the lysis treatment, 3 M sodium acetate was added and followed by the addition of phenol:chloroform:isoamyl alcohol, pH 7.8. The solution was centrifuged at 16k x g for 5 min. After centrifugation, the aqueous fraction was isolated and re-treated with a chloroform:isoamyl alcohol solution to remove residual phenol. The centrifugation and aqueous layer removal was repeated. The aqueous fraction was then treated with 100 % ice-cold ethanol and the DNA was precipitated overnight at −80 °C. After the overnight step, the DNA precipitate was centrifuged at 16k x g at 4 °C for 30 min and the supernatant was removed. The DNA was resuspended in 200 µL of dH_2_O and the precipitation protocol was repeated to ensure the complete removal of SDS from the initial lysis step. After completing the second precipitation, the DNA pellet was dried in a Speed-Vac for 10 min without heating. The dried DNA pellet was gently resuspended in 200 µL dH_2_O, quantitated by NanoDrop, and stored at −80 °C .

### 4.2 Sequencing

0.21 µg of total extracted DNA was sent to Beijing Genomic Institute (BGI) for NGS. BGI performed the library preparation by utilizing their Optimal DNA Library Prep Kit which included a PCR amplification step. The library was composed of paired end 150 base pair reads, that was sequenced on a MGISEQ-2000. Following data collection, BGI cleaned the raw data with the SOAPnuke program [13], removing non-determinative reads with more than 1 % N content, adapter sequences, sequences less than 150 bp in length, and sequences with a quality score of less than 20. The cleaned raw data file was then transferred to FfAME for analysis and assembly. The raw data file metrics for AK85 are listed in Table 1.

### 4.3 Assembly and functional annotation

Raw read files were assessed with FastQC to determine the quality of the sequenced and cleaned reads provided by BGI [14]. The FastQC analysis indicated a dataset that contained zero N bases, adapter content, overrepresented sequences, and an average quality score of 39. The genome was then assembled with SKESA assembler with default parameters [15]. The SKESA assembly file was then analyzed by QUAST, CheckM, and BUSCO [16,17]. The SKESA fasta file was uploaded to Proskee for genome map visualization [5]. Additionally, AK85’s CDS regions were annotated with Prokka, antibiotic resistance genes identified with CARD, and the ANI was calculated with FastANI against the *T. uzonensis* genome available on NCBI [[6], [7], [8]]. All of the aforementioned steps were conducted within Proskee.

### 4.4 16S rRNA phylogenetic analysis

All 16S rRNA sequences for the phylogenetic comparison were retrieved from NCBI. AK85’s 16S rRNA partial sequence was manually retrieved from the skesa assembly output file. All of the sequences were uploaded to https://ngphylogeny.fr/ for alignment and phylogenetic tree construction [18]. The 16S rRNA sequences were aligned with MAFFT (v 7.407), alignments were curated with BMGE (v1.12), and the phylogenetic tree was constructed with 1000 bootstrap replicates using FastME (v2.1.6.1). The phylogenetic tree was visualized and exported with iTOL [19].

### 4.5 Alcohol dehydrogenase activity screen

Intracellular NAD^+^ and NADP^+^ dependent ADH activities of AK85 were assessed colorimetrically based on Fibla & Gonzales-Duarte [20]. All alcohol substrates assayed were obtained from Sigma-Aldrich with a purity of min. 98 % (with the exception of (R) 1,2-propanediol and (S) 1,2-propanediol which were 96 %). An overnight 1L culture was used to inoculate BM medium supplemented with 20 mM glucose for 18 h at 65 °C in a single 1 L serum bottle with a 1:1 liquid-to-gas ratio without shaking. Following incubation, cells were harvested by centrifugation at 3800 x g for 15 min and resuspended in 30 mL of 50 mM Tris-HCl pH 8.5.

The cell pellets were lysed by bead beating using 150–212 µm glass beads in a 1:1 ratio (bead weight to wet cell weight) and then vortexed, alternating with cooling on ice between cycles. Each cycle was 15 s of vortexing followed by 30 s of cooling, repeated 10–15 times. The resulting cell lysates were centrifuged and the supernatant assayed for the corresponding activity of ADHs in technical triplicate. The following solutions were combined in a Honeycomb microplate well: 50 µL cell lysate, 122 µL reagent solution (0.217 g NAD^+^ or 0.243 g NADP^+^, 0.135 g 3-(4,5-dimethylthiazol-2-yl)-2,5-diphenyltetrazolium bromide (MTT), and 1.3 g gelatin in 900 mL 50 mM Tris-HCl, pH 8.5), 13.5 µL of 74 mM solution of alcohol of interest and 15 µL of phenazine methosulfate (PMS) solution (0.245 g/L PMS in 50 mM Tris-HCl, pH 8.5), bringing the final assay reagents concentrations to: 200 µM cofactor of interest, 200 µM MTT, 0.9 g/L gelatin, 5.0 mM alcohol of interest, and 60 µM PMS.

The assay mixture was incubated at room temperature for 10 min after which an increase in absorbance was monitored at 590 nm at 59 °C for 1 h every 10 min using a Bioscreen C (GrowthCurves, Ltd, Finland) microplate reader. Standard curve was prepared using NADH in the range of 0–50 nmol and used to calculate volumetric activity (mL).

## Limitations

The Alcohol Dehydrogrenase Activity Screen has two limitations. Specifically, the data solely pertains to AK85 wild-type lysates generated from growth in BM media, without protein concentration assessments. Further, there is no data pertaining to individual alcohol dehydrogenase enzymes from knockout-variants or purified enzymes from lysate for analysis.

## Ethics Statement

The research detailed in this manuscript did not involve studies with animals or humans. Therefore, we confirm that our research adheres to the ethical requirements for publication in Data in Brief.

## Credit Author Statement

**Clay A. Abraham:** Investigation, Visualization, Writing- Original Draft, Writing- Review, Editing. **Eva Maria Ingvadottir:** Investigation, Writing- Original Draft, Writing- Review, Editing. **Kevin M. Bradley:** Methodology, Software, Writing – review & editing. **Sean M. Scully:** Conceptualization, Supervision, Methodology, Writing – review & editing. **Johann Orlygsson:** Conceptualization, Writing – review & editing. **Derek Dube:** Conceptualization, Supervision, Methodology, Writing – review & editing. **Steven A. Benner:** Supervision and Financial Support

## Data Availability

NCBIThermoanaerobacter uzonensis strain:AK85 Genome sequencing (Original data)
Mendeley DataThermoanaerobacter uzonensis strain AK85 ADH Lysate Activity Screen and Phylogenetic Data (Original data)

## Appendix Supplementary materials

Image, application 1
