# Supplementary material for: Draft genome and alcohol dehydrogenase dataset of thermoanaerobacter uzonensis bacterium strain AK85
Source: Data Brief. 2025 Oct 27;63:112192. doi: 10.1016/j.dib.2025.112192 (PMC12603687; doi:10.1016/j.dib.2025.112192)
Supplement: Supplementary file 1 [file mmc1.docx]

| Supplementary Table 1.1 *T. uzonensis* strain AK85 Carbohydrate Annotations | | | |
| --- | --- | --- | --- |
| **Enzyme** | **Length (bp)** | **Gene Name** | **EC_number** |
| **Carbohydrate Utilization** | | | |
| Cellobiose 2-epimerase | 1143 | *-* | 5.1.3.11 |
| Cellobiose phosphorylase | 2388 | *cbpA* | 2.4.1.20 |
| PTS system cellobiose-specific EIIB component | 312 | *celA* | 2.7.1.205 |
| Lactose transport system permease protein LacF | 879 | *lacF_1* |  |
| Galactokinase | 1164 | *galK* | 2.7.1.6 |
| Galactose/methyl galactoside import ATP-binding protein MglA | 1530 | *mglA_1* | 7.5.2.11 |
| PTS system galactitol-specific EIIC component | 1248 | *gatC_2* |  |
| PTS system galactitol-specific EIIB component | 294 | *gatB_2* | 2.7.1.200 |
| Galactose-6-phosphate isomerase subunit LacB | 909 | *lacB* | 5.3.1.26 |
| Beta-galactosidase | 2076 | *lacZ_1* | 3.2.1.23 |
| Alpha-galactosidase AgaA | 2202 | *agaA* | 3.2.1.22 |
| Xylose transport system permease protein XylH | 1191 | *xylH_1* |  |
| Xylose import ATP-binding protein XylG | 1524 | *xylG_1* | 7.5.2.10 |
| D-xylose-binding periplasmic protein | 1080 | *xylF_1* |  |
| Xylose transport system permease protein XylH | 1164 | *xylH_2* |  |
| Xylose import ATP-binding protein XylG | 1536 | *xylG_2* | 7.5.2.10 |
| D-xylose-binding periplasmic protein | 1110 | *xylF_2* |  |
| Xylulose kinase | 1503 | *xylB_1* | 2.7.1.17 |
| Xylose isomerase | 1314 | *xylA* | 5.3.1.5 |
| Mannitol-1-phosphate 5-dehydrogenase | 1167 | *mtlD_2* | 1.1.1.17 |
| Mannitol-specific phosphotransferase enzyme IIA component | 441 | *mtlF* |  |
| PTS system mannitol-specific EIICB component | 1437 | *mtlA* |  |
| Mannitol-1-phosphate 5-dehydrogenase | 1215 | *mtlD_1* | 1.1.1.17 |
| PTS system sorbose-specific EIIB component | 504 | *sorB* | 2.7.1.206 |
| PTS system sorbose-specific EIIC component | 750 | *sorC* |  |
| Trehalose transport system permease protein SugB | 834 | *sugB* |  |
| Trehalose transport system permease protein SugA | 924 | *sugA_1* |  |
| Trehalose transport system permease protein SugA | 855 | *sugA_2* |  |
| Alpha,alpha-trehalose phosphorylase | 2343 | *treP* | 2.4.1.64 |
| Glucoamylase | 2115 | *cga* | 3.2.1.3 |
| Glucose--fructose oxidoreductase | 1089 | *gfo* | 1.1.99.28 |
| Glucose-6-phosphate isomerase B | 1356 | *pgiB* | 5.3.1.9 |
| Glucose-6-phosphate 1-dehydrogenase | 1458 | *zwf* | 1.1.1.49 |
| PTS system glucose-specific EIIA component | 483 | *crr_2* |  |
| PTS system glucose-specific EIIA component | 486 | *crr_1* |  |
| Multiple sugar-binding periplasmic receptor ChvE | 1146 | *chvE* |  |
| Maltogenic alpha-amylase | 1581 | *amyM* | 3.2.1.133 |
| Maltose/maltodextrin transport system permease protein MalG | 894 | *malG* |  |
| Maltose/maltodextrin transport system permease protein MalF | 921 | *malF* |  |
| Maltose/maltodextrin-binding periplasmic protein | 1245 | *malE* |  |
| Alpha-maltose-1-phosphate synthase | 1167 | *glgM* | 2.4.1.342 |

| Supplementary Table 1.2 *T. uzonensis* strain AK85 Annotations | | | |
| --- | --- | --- | --- |
| **Enzyme** | **Length (bp)** | **Gene Name** | **EC_number** |
| ***Energy Production*** | | | |
| L-lactate dehydrogenase | 936 | *ldh_2* | 1.1.1.27 |
| Pyruvate:ferredoxin oxidoreductase | 3513 | *-* | 1.2.7.1 |
| Pyruvate:ferredoxin oxidoreductase | 3528 | *-* | 1.2.7.1 |
| Acetate kinase | 1206 | *ackA* | 2.7.2.1 |
| NADH oxidase | 1947 | *-* |  |
| Adenylate kinase | 654 | *adk* | 2.7.4.3 |
| Isocitrate dehydrogenase | 1212 | *icd* | 1.1.1.42 |
| Pyruvate synthase subunit PorC | 558 | *porC* | 1.2.7.1 |
| 2-oxoglutarate oxidoreductase subunit KorA | 1068 | *korA_1* | 1.2.7.3 |
| 2-oxoglutarate oxidoreductase subunit KorA | 1680 | *korA_2* | 1.2.7.3 |
| 2-oxoglutarate oxidoreductase subunit KorA | 1125 | *korA_3* | 1.2.7.3 |
| 2-oxoglutarate oxidoreductase subunit KorA | 1128 | *korA_4* | 1.2.7.3 |
| 2-oxoglutarate oxidoreductase subunit KorB | 750 | *korB* | 1.2.7.3 |
| Pyrophosphate--fructose 6-phosphate 1-phosphotransferase | 1239 | *pfp* | 2.7.1.90 |
| Fructose-bisphosphate aldolase | 972 | *fba_1* | 4.1.2.13 |
| Fructose-bisphosphate aldolase | 852 | *fba_2* | 4.1.2.13 |
| Phosphoenolpyruvate-protein phosphotransferase | 1722 | *ptsI* | 2.7.3.9 |
| Pyruvate, phosphate dikinase | 2634 | *ppdK* | 2.7.9.1 |
| Potassium-transporting ATPase KdpC subunit | 579 | *kdpC* |  |
| Potassium-transporting ATPase ATP-binding subunit | 2040 | *kdpB* | 7.2.2.6 |
| Potassium-transporting ATPase potassium-binding subunit | 1686 | *kdpA* |  |
| ***Broad Amino Acids*** | | | |
| Branched-chain-amino-acid aminotransferase | 876 | *ilvE* | 2.6.1.42 |
| High-affinity branched-chain amino acid transport ATP-binding protein LivF | 720 | *livF_2* | - |
| High-affinity branched-chain amino acid transport system permease protein LivH | 867 | *livH* | - |
| High-affinity branched-chain amino acid transport ATP-binding protein LivF | 729 | *livF_1* | - |
| putative amino acid permease YhdG | 1410 | *yhdG_1* | - |
| putative amino acid permease YhdG | 1380 | *yhdG_2* | - |
| **Esterases & Lipases** | | | |
| Protein-glutamate methylesterase/protein-glutamine glutaminase | 585 | *cheB_2* | 3.1.1.61 |
| Pimeloyl-[acyl-carrier protein] methyl ester esterase | 897 | *bioH* | 3.1.1.85 |
| Monoacylglycerol lipase | 942 | *-* | 3.1.1.23 |
| Monoacylglycerol lipase | 942 | *-* | 3.1.1.23 |
| **Sulfur Cycling** | | | |
| Homoserine O-acetyltransferase | 1101 | *metXA* | 2.3.1.31 |
| 5-methyltetrahydropteroyltriglutamate--homocysteine methyltransferase | 2295 | *metE* | 2.1.1.14 |
| L-methionine gamma-lyase | 1191 | *mgl_1* | 4.4.1.11 |
| L-methionine gamma-lyase | 1191 | *mgl_2* | 4.4.1.11 |
| O-acetyl-L-homoserine sulfhydrylase | 1284 | *-* | 2.5.1.- |
| Cysteine synthase | 936 | *cysK_1* | 2.5.1.47 |
| Cysteine desulfurase SufS | 1143 | *sufS_1* | 2.8.1.7 |
| Serine acetyltransferase | 666 | *cysE* | 2.3.1.30 |
| Precorrin-2 dehydrogenase | 573 | *sirC* | 1.3.1.76 |
| Siroheme synthase | 1494 | *cysG* | 4.99.1.4 |
| Sirohydrochlorin ferrochelatase | 366 | *sirB* | 4.99.1.4 |
